# LightSpot Fluorescent Conjugates as Highly Efficient Tools for Lysosomal P-gp Quantification in Olaparib-Treated Triple-Negative Breast Cancer Cells

**DOI:** 10.3390/ijms26146675

**Published:** 2025-07-11

**Authors:** Antoine Goisnard, Pierre Daumar, Maxime Dubois, Elodie Gay, Manon Roux, Marie Depresle, Frédérique Penault-Llorca, Emmanuelle Mounetou, Mahchid Bamdad

**Affiliations:** 1 UMR INSERM-UCA, U1240, Imagerie Moléculaire et Stratégies Théranostiques, Institut Universitaire de Technologie, Université Clermont Auvergne, F-63000 Clermont Ferrand, France; antoine.goisnard@uca.fr (A.G.); pierre.daumar@uca.fr (P.D.); maxime.dubois@uca.fr (M.D.); elodie.gay@uca.fr (E.G.); manon.roux@uca.fr (M.R.); marie.depresle@uca.fr (M.D.); emmanuelle.mounetou@inserm.fr (E.M.); 2 UMR INSERM-UCA, U1240, Imagerie Moléculaire et Stratégies Théranostiques, Centre de Lutte Contre le Cancer Jean Perrin, Université Clermont Auvergne, F-63000 Clermont Ferrand, France; frederique.penault-llorca@clermont.unicancer.fr

**Keywords:** multidrug resistance, permeability-glycoprotein, lysosomal P-gp, LightSpot fluorescent conjugates, triple-negative breast cancer cell lines

## Abstract

P-glycoprotein (P-gp) is a key element of cancer treatment resistance, actively extruding cytotoxic drugs from cells and diminishing their efficacy. While its role at the plasma membrane is well established, its intracellular localization, particularly on lysosomes, is increasingly recognized as a critical contributor to drug resistance. This study investigates four innovative LightSpot fluorescent compounds to detect and quantify both membrane and lysosomal P-gp in Triple-Negative Breast Cancer (TNBC) SUM1315 and DU4475 cell lines. Results highlighted lysosomal P-gp staining by the LightSpot-FL-1, LightSpot-BrX-1, and LightSpot-BdO-1 fluorescent compounds (Mander’s coefficients > 0.8 overlapping with LAMP2 immunostaining). After both cell lines were exposed to Olaparib, a significant increase in P-gp expression level and lysosomal distribution of P-gp was detected. Indeed, after 100 µM Olaparib exposure, LightSpot-FL-1 allowed us to quantify an increase in P-gp-positive lysosome number of 1293 and 334% for SUM1315 and DU4475 cells, respectively, compared to the control. Findings suggest that P-gp may relocate to lysosomes upon drug exposure, highlighting a dual resistance mechanism involving both membrane and lysosomal P-gp. This study demonstrated the potential of LightSpot fluorescent compounds to evaluate P-gp-mediated cell resistance to treatment and emphasized the need to assess global cell P-gp expression to improve cancer diagnosis.

## 1. Introduction

Multidrug resistance (MDR) represents a major challenge in tumor treatment and refers to a complex phenomenon resulting from the contribution of various biological mechanisms [1]. Among these mechanisms, drug efflux mediated by ATP-binding cassette (ABC) transporters represents the first line of cellular defense and is supported by multiple membrane proteins, such as the well-studied P-glycoprotein (P-gp) [2,3]. Indeed, P-gp is a transmembrane protein that acts as an efflux pump and is involved in the transport of various xenobiotics across cell membranes, including a large panel of anticancer drugs [2]. In this way, this transporter prevents drugs from reaching their target, thus reducing treatment efficacy. Its important role in tumor resistance has been studied for many years in order to optimize anticancer treatments [2,3]. Several studies have reported a high P-gp expression level in many cancer types, with a strong correlation with negative clinical outcomes such as treatment failure, relapse, and low rate of survival [4,5,6,7,8].

Despite the evident pertinence of contemplating P-gp as a tumor resistance biomarker and a therapeutic target in the field of oncology, the clinical investigation of this protein remains quite limited, both in terms of diagnosis and therapeutics. This can be mainly explained by the lack of a standardized, efficient, and clinically validated methodology allowing the quantification and localization of the P-gp in tumor cells [3,9]. Several methodological and specificity limitations associated with antibody-based assays, the most classic approach used in this context, were reported [10]. Indeed, the detection of P-gp by immunohistochemistry presents a great variability between studies, leading to contradictory and unclear results [9,11,12].

Otherwise, several works demonstrated that P-gp was not only distributed on plasma membranes, but also on membranes of organelles such as mitochondria and intracellular vesicles of the endo-lysosomal system [13,14,15]. If mitochondrial P-gp localization remains uncertain, that on lysosome membranes is clearly established [13,15,16]. Several studies showed that this lysosomal P-gp confers MDR through drug sequestration in the intra-lysosomal compartment [14,17]. For this, lysosomal P-gp promotes the trapping of various chemotherapeutics within lysosomes, preventing them from reaching their target [18]. Moreover, acidic intra-lysosomal pH induces ionization of some therapeutic agents, making them unable to cross the lysosome membrane, and facilitates their degradation [14,18]. These findings demonstrated the importance of considering lysosomal P-gp, as well as cell membrane P-gp, for a better investigation of MDR in cancers [14,18]. As a consequence, new strategies were recently investigated aiming to target the lysosomal P-gp.

In this context, our team has developed a series of P-gp-targeted fluorescent conjugates entitled LightSpot as an alternative method to detect, quantify, and localize the P-gp in biological samples [19,20]. These small molecules with a peptidic scaffold coupled with a fluorochrome present the ability to cross the cell membranes. Thus, LightSpot compounds are able to detect and quantify global P-gp located on cell membranes and organelle membranes. Various pharmacomodulation strategies have led to the design, the synthesis, and the evaluation of 24 P-gp-targeted LightSpot fluorescent conjugates with different structures and optical properties [19]. Due to their lipophilicity, these fluorescent conjugates present the ability to penetrate cells and reach intracellular compartments to target the P-gp in cells. Thus, total cell P-gp expressed at the cell membrane and organelle levels may be measured by these tools [21]. Interestingly, P-gp staining detected with these compounds was localized both on cell membranes and intracellular vesicle-like structures [19].

For the biological evaluation of these P-gp-targeted fluorescent conjugates, a Triple-Negative Breast Cancer (TNBC) model was selected. This immunohistochemical breast cancer subtype is characterized by the absence of significant estrogen and progesterone receptor expression and the lack of human epidermal receptor 2 (HER2) overexpression/amplification and thus presents a poor prognosis [11]. Moreover, several studies focused on TNBC demonstrated that the expression of P-gp represents a real obstacle in treatment outcomes [9,22,23] and may be a useful marker of metastatic spread [24]. Indeed, our previous works clearly demonstrated the efficiency of one of these fluorescent compounds, entitled LightSpot-FL-1, for the detection and quantification of P-gp expression level. Increased P-gp expression level was found in the presence of anticancer drugs such as Olaparib (OLA) on several cancer monolayer cell cultures and spheroid TNBC models [21].

In this context, the present study aims to investigate more deeply the P-gp staining ability of four fluorescent compounds, i.e., LightSpot-FL-1, LightSpot-BrX-1, LightSpot-Scy-1, and LightSpot-BdO-1, in order to explore their efficacy to target membrane and lysosomal P-gp in two SUM1315 and DU4475 TNBC cell line models. Based on P-gp silencing using siRNAs and fluorescence imaging, the specificity of the compounds for the specific detection of the P-gp was firstly evaluated. Then, subcellular distribution of fluorescent conjugate staining was studied through organelle co-staining experiments. Finally, cellular and lysosomal P-gp response after increased OLA cell exposure was explored.

## 2. Results

### 2.1. LightSpot Fluorescent Conjugate Intracellular Staining Distribution in SUM1315 and DU4475 Cell Lines

**Figure 1:**
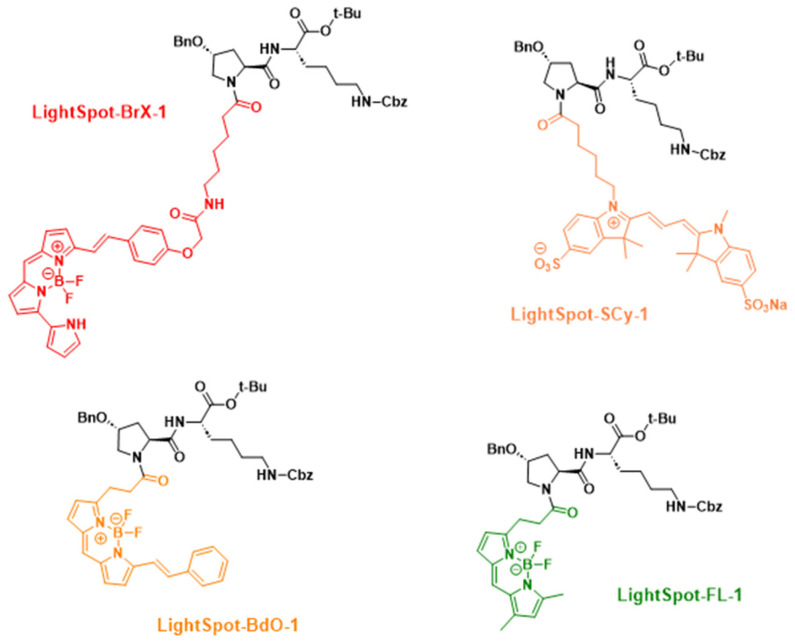
Chemical structures of conjugates LightSpot-FL-1, LightSpot-BrX-1, LightSpot-Scy-1, and LightSpot-BdO-1. Bn, Cbz, and t-Bu abbreviations on the black peptidic scaffold represent benzyl, carbobenzyloxy, and tert-butyl groups, respectively. Fluorophores are shown in color.

The intracellular staining distribution by the four LightSpot fluorescent conjugates LightSpot-FL-1, LightSpot-BrX-1, LightSpot-Scy-1, and LightSpot-BdO-1 (Figure 1) was investigated using TNBC SUM1315 and DU4475 cell models. Lysosomal and mitochondrial immunostaining was also realized using anti-LAMP2 and anti-ATP5A antibodies, respectively. Parallelly, comparison of LightSpot compound staining using Hoechst33258 was carried out in order to control the absence of a fluorescent signal in the nuclear compartment. For each fluorescent conjugate, a colocalization study was conducted, including Pearson’s correlation and Mander’s overlap coefficient analyses.

**Figure 2:**
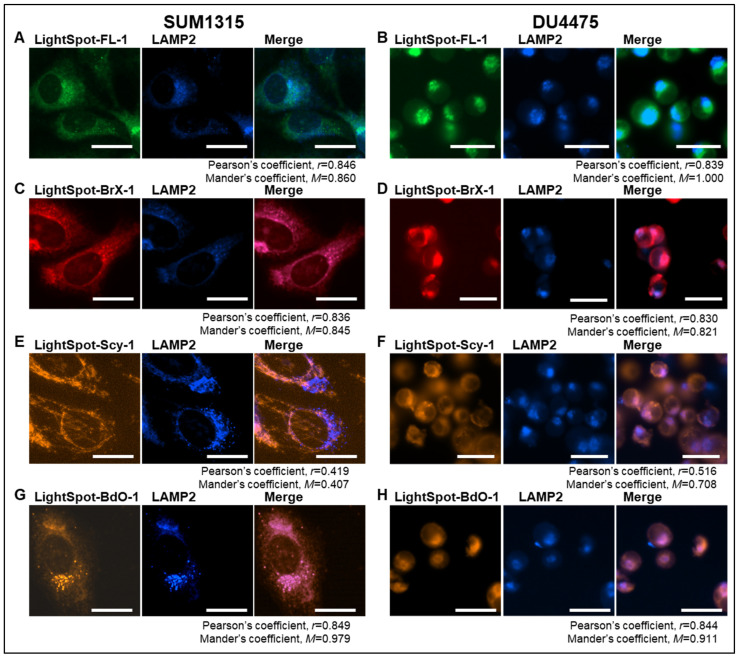
Colocalization study between LightSpot fluorescent conjugates and lysosomal stainings. Cellular co-staining combining LightSpot-FL-1 (**A**,**B**), LightSpot-BrX-1 (**C**,**D**), LightSpot-Scy-1 (**E**,**F**), and LightSpot-BdO-1 (**G**,**H**), and anti-LAMP2 immunostaining, were realized in SUM1315 (**A**,**C**,**E**,**F**) and DU4475 (**B**,**D**,**E**,**H**) cells. For each case, merged images are presented next to the LightSpot and anti-LAMP2 staining images. Images were acquired with a Cytation™5MV multimodal plate reader (BioTek^®^, M = 40x, DAPI, GFP, Cy5, and RFP filters, scale bar = 20 µm). Pearson’s correlation (r) and Mander’s overlap (M) coefficients were calculated for each co-staining and are presented below images.

**Figure 3:**
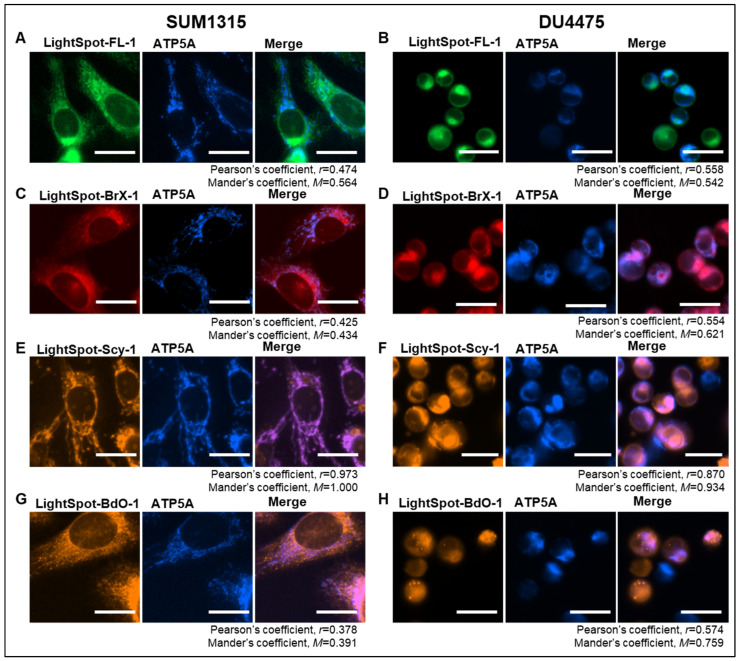
Colocalization study between LightSpot fluorescent conjugates and mitochondrial stainings. Cellular co-staining combining LightSpot-FL-1 (**A**,**B**), LightSpot-BrX-1 (**C**,**D**), LightSpot-Scy-1 (**E**,**F**), and LightSpot-BdO-1 (**G**,**H**), and anti-ATP5A immunostaining, were realized in SUM1315 (**A**,**C**,**E**,**F**) and DU4475 (**B**,**D**,**E**,**H**) cells. For each case, merged images are presented next to the LightSpot and anti-ATP5a staining images. Images were acquired with a Cytation™5MV multimodal plate reader (BioTek^®^, M = 40x, DAPI, GFP, Cy5, and RFP filters, scale bar = 20 µm). Pearson’s correlation (r), and Mander’s overlap (M) coefficients were calculated for each co-staining and are presented below images.

**Figure 4:**
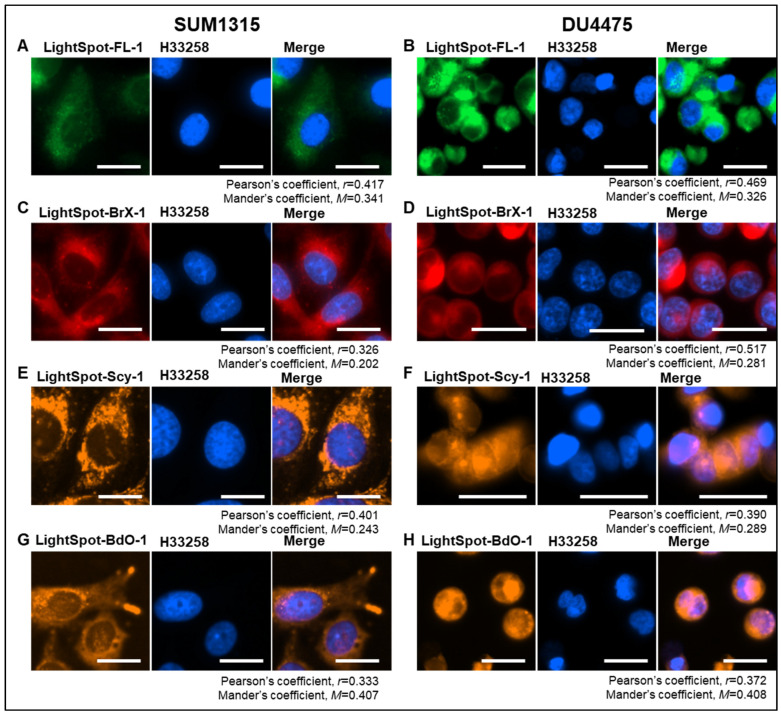
Colocalization study between LightSpot fluorescent conjugate staining and nuclear staining. Cellular co-staining combining LightSpot-FL-1 (**A**,**B**), LightSpot-BrX-1 (**C**,**D**), LightSpot-Scy-1 (**E**,**F**), and LightSpot-BdO-1 (**G**,**H**), and Hoechst 33,258 (H33258) nuclear staining were realized in SUM1315 (**A**,**C**,**E**,**F**) and DU4475 (**B**,**D**,**E**,**H**) cells. For each case, merged images are presented next to the LightSpot and H33258 staining images. Images were acquired with a Cytation™5MV multimodal plate reader (BioTek^®^, M = 40x, DAPI, GFP, Cy5, and RFP filters, scale bar = 20 µm). Pearson’s correlation (r), and Mander’s overlap (M) coefficients were calculated for each co-staining and are presented below images.

Firstly, for the LightSpot-FL-1 conjugate, a strong correlation with LAMP2 lysosomal immunostaining was detected, with a Pearson’s coefficient of 0.846 and a Mander’s coefficient of 0.860 in SUM1315 cells (Figure 2A), and a Pearson’s coefficient of 0.839 and a Mander’s coefficient of 1.000 in DU4475 cells (Figure 2B). In contrast, low correlation with anti-ATP5A mitochondrial immunostaining was noted in both cell lines, with a Pearson’s coefficient equal to 0.474 and a Mander’s coefficient of 0.564 in SUM1315 cells (Figure 3A), and a Pearson’s coefficient of 0.558 and a Mander’s coefficient of 0.542 for DU4475 cells (Figure 3B). Likewise, low correlation with nuclear staining was quantified with a Pearson’s coefficient of 0.417 and a Mander’s coefficient of 0.341 in SUM1315 cells (Figure 4A), and a Pearson’s coefficient of 0.469 and a Mander’s coefficient of 0.326 in DU4475 cells (Figure 4B).

Secondly, for LightSpot-BrX-1 conjugate, a strong correlation with lysosomal immunostaining in SUM1315 cells with a Pearson’s coefficient equal to 0.836 and a Mander’s coefficient of 0.845 (Figure 2C), and in DU4475 cells with a Pearson’s coefficient of 0.830 and a Mander’s coefficient of 0.821 (Figure 2D) was detected. A low correlation was detected, though, with anti-ATP5A mitochondrial immunostaining in SUM1315 cells, with Pearson’s coefficient = 0.425 and Mander’s coefficient = 0.434 (Figure 3C), and for DU4475 cells, with Pearson’s coefficient = 0.554 and Mander’s coefficient = 0.621 (Figure 3D). In the same way, low correlation with nuclei was detected in SUM1315 cells with Pearson’s coefficient = 0.326 and Mander’s coefficient = 0.202 (Figure 4C) and in DU4475 cells with Pearson’s coefficient = 0.517 and Mander’s coefficient = 0.281 (Figure 4D).

Thirdly, for LightSpot-SCy-1 conjugate, contrary to the two previous compounds, low correlation was detected with LAMP2 lysosomal immunostaining in SUM1315 cells with a Pearson’s coefficient = 0.419 and a Mander’s coefficient = 0.407 (Figure 2E), and in DU4475 cells with a Pearson’s coefficient = 0.516 and a Mander’s coefficient = 0.911 (Figure 2F). However, strong correlation was obtained with anti-ATP5A mitochondria immunostaining in SUM1315 cells with a Pearson’s coefficient of 0.973 and a Mander’s coefficient of 1.000 (Figure 3E), and in DU4475 cells with a Pearson’s coefficient of 0.870 and a Mander’s coefficient of 0.934 (Figure 3F). Otherwise, low correlation was noted with nuclear staining in SUM1315 cells with a Pearson’s coefficient of 0.401 and a Mander’s coefficient of 0.243 (Figure 3E), and in DU4475 cells with a Pearson’s coefficient of 0.390 and a Mander’s coefficient of 0.289 (Figure 3F).

Finally, for the LightSpot-BdO-1 compound, a strong correlation was quantified with anti-LAMP2 lysosomal immunostaining in SUM1315 cells with a Pearson’s coefficient of 0.849 and a Mander’s coefficient of 0.979 (Figure 2G), and in DU4475 cells with a Pearson’s coefficient of 0.844 and a Mander’s coefficient of 0.911 (Figure 2H). Low correlation was measured with anti-ATP5A mitochondrial immunostaining in SUM1315 cells with a Pearson’s coefficient of 0.378 and a Mander’s coefficient of 0.391 (Figure 3G) and DU4475 cells with a Pearson’s coefficient of 0.574 and a Mander’s coefficient of 0.759 (Figure 3H). Similarly, low correlation with nuclei was measured in SUM1315 cells with a Pearson’s coefficient of 0.333 and a Mander’s coefficient of 0.407 (Figure 4G), and in DU4475 cells with a Pearson’s coefficient of 0.372 and a Mander’s coefficient of 0.408 (Figure 4H).

These results demonstrate lysosomal intracellular vesicle staining by the three LightSpot fluorescent compounds bearing a BODIPY fluorophore, i.e., LightSpot-FL-1, LightSpot-BrX-1, and LightSpot-BdO-1. In contrast, the sulfocyanine-based conjugate LightSpot-Scy-1 presents a different filamentous staining pattern and seems to accumulate at the mitochondrial level.

### 2.2. Selective P-gp Targeting Efficiency Study by the Four LightSpot Fluorescent Conjugates in SUM1315 and DU4475 Cell Lines

To identify LightSpot fluorescent conjugates able to target the P-gp, an evaluation of the specificity of these compounds staining was conducted. For this, P-gp knockdown cell models derived from SUM1315 and DU4475 cell lines were developed using P-gp siRNAs.

In order to validate the efficiency of P-gp knockdown, an anti-P-gp immunostaining was carried out in both SUM1315 and DU4475 cell lines 72 h after transfection with siRNAs. In SUM1315, a high extinction of immunostaining with 2.7 ± 0.6 × 10^3^ Fluorescence Units (FU) was detected in comparison to control cells with 12.7 ± 2.0 × 10^3^ FU (*p* < 0.0001) (Figure S1A,B). In the same way, in DU4475 cells, a significant decrease in fluorescent signal was measured in P-gp siRNA-treated cells with 16.2 ± 3.2 × 10^3^ FU, in comparison to control cells with 26.2 ± 4.0 × 10^3^ FU (*p* < 0.0001) (Figure S1A,C).

In addition, the selectivity of the P-gp silencing was also controlled in the same experimental conditions, using an anti-BCRP (ABCG2) and an anti-MRP1 (ABCC1) immunostaining, i.e., two other MDR proteins. With BCRP immunostaining, no significant differences were detected between P-gp knockdown cells and non-treated cells in both SUM1315 and DU4475 cell lines (Figure S1D). Indeed, in the SUM1315 cell line, siRNA-treated cells presented BCRP staining fluorescence intensity of 10.4 ± 0.3 × 10^3^ FU that was comparable to 10.4 ± 0.2 × 10^3^ FU (*p* = 0.9999) for untreated cells (Figure S1E). Likewise, DU4475 siRNA-treated cells showed a fluorescence intensity of BCRP staining of 10.1 ± 0.3 × 10^3^ FU, comparable to 10.3 ± 1.9 × 10^3^ FU (*p* = 0.8793) for control cells (Figure S1F). In the same way, after P-gp silencing, no differences in anti-MRP1 immunostaining were observed in SUM1315 and DU4475 cells in comparison to the control (Figure S1G). In the SUM1315 cell line, transfected cells presented a fluorescence intensity equal to 10.1 ± 0.3 × 10^3^ FU, similar to 10.3 ± 1.9 × 10^3^ FU (*p* = 0.8237) for control cells (Figure S1H), and in the DU4475 cell line, transfected cells presented a fluorescence intensity of 11.3 ± 0.9 × 10^3^ FU, similar to 11.5 ± 0.7 × 10^3^ FU (*p* = 0.8237) for control cells (Figure S1I). All these results allowed us to validate two cell models presenting a very highly silenced P-gp expression, with no alteration of other major MDR proteins. These models were thus used thereafter as negative P-gp expression cells to evaluate the targeting of this protein by the four LightSpot fluorescent conjugates.

**Figure 5:**
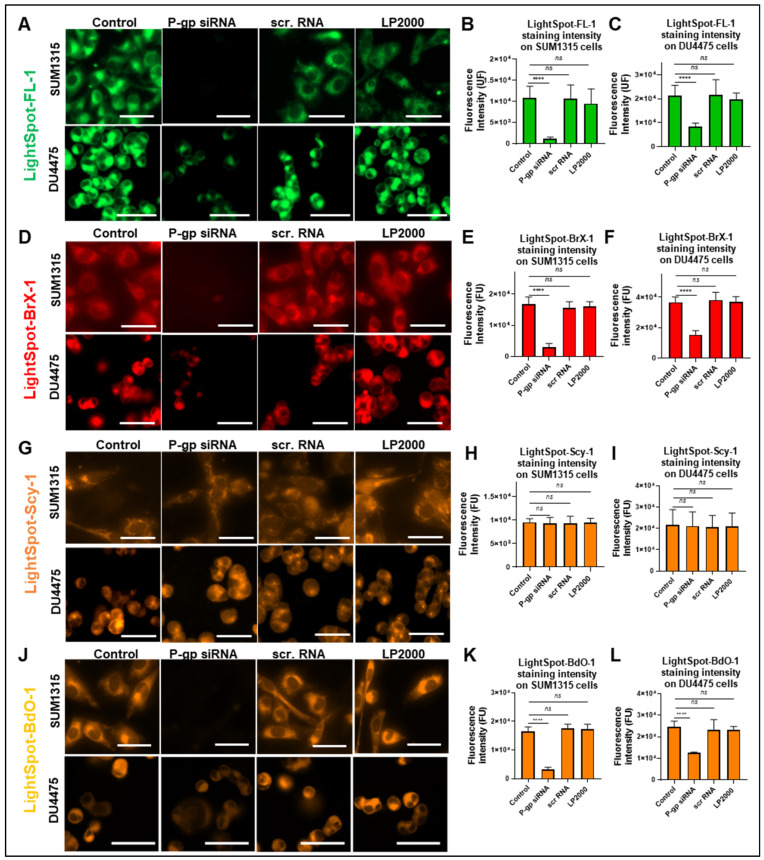
Validation of LightSpot fluorescent conjugate specificity using P-gp siRNAs in TNBC SUM1315 and DU4475 cell lines. To evaluate the specificity of the 4 conjugates, cells were exposed for 72 h to either no additional reagent (control), P-gp siRNAs, scrambled RNAs (scr RNA), or Lipofectamine2000 alone (LP2000). After PFA fixation, SUM1315 and DU4475 cells were stained with LightSpot-FL-1 (**A**), LightSpot-BrX-1 (**D**), LightSpot-Scy-1 (**G**), or LightSpot-BdO-1 (**J**) conjugates and imaged with Cytation™5MV (BioTek^®^, M = 40x, GFP, Cy5, and RFP filters, scale bar = 50 µm). Mean cellular fluorescence intensity on images was quantified using Gen5 software (BioTek^®^) in SUM1315 cells after LightSpot-FL-1 (**B**), LightSpot-BrX-1 (**E**), LightSpot-Scy-1 (**H**), or LightSpot-BdO-1 (**K**) staining and in DU4475 cells after LightSpot-FL-1 (**C**), LightSpot-BrX-1 (**F**), LightSpot-Scy-1 (**I**), or LightSpot-BdO-1 (**L**) staining. Data are presented on graphs as mean ± SD. Significance was determined by one-way ANOVA, ns *p* > 0.05, **** *p* < 0.0001.

For the evaluation of LightSpot-FL-1 conjugate specificity, a complete extinction of fluorescence signal was observed in P-gp siRNA-transfected SUM1315 cells with 1.2 ± 0.4 × 10^3^ FU, in comparison to the three evaluated controls that were untreated control cells with 10.8 ± 2.7 × 10^3^ FU, cells treated with scrambled (scr) RNAs with 10.6 ± 3.2 × 10^3^ FU, or cells treated with Lipofectamine2000 (LP2000) alone with 9.5 ± 3.5 × 10^3^ FU (Figure 5A,B) (*p* < 0.0001, respectively). In DU4475 (Figure 5A), P-gp silenced cells presented a fluorescence intensity of 8.4 ± 1.5 × 10^3^ FU, significantly inferior to 21.3 ± 4.3 × 10^3^, 21.6 ± 6.3 × 10^3^, and 19.6 ± 2.7 × 10^3^ FU measured for untreated control, scrRNAs, and LP2000 conditions, respectively (*p* < 0.0001) (Figure 5C). In this cell line, a partial but important reduction in fluorescence signal intensity was detected in P-gp knockdown cells in comparison to control conditions.

Regarding the specificity of LightSpot^®^-BrX-1 conjugate staining, in the SUM1315 cell line, P-gp silenced cells presented a fluorescence intensity of 2.5 ± 0.5 × 10^3^ FU, significantly inferior to 16.7 ± 2.3 × 10^3^, 15.5 ± 2.0 × 10^3^, and 16.0 ± 1.5 × 10^3^ FU measured for untreated, scrRNAs, and LP2000 control conditions, respectively (*p* < 0.0001) (Figure 5D,E). Otherwise, in DU4475 cells, P-gp knockdown cells presented lower LightSpot-BrX-1 staining intensity with 15.3 ± 2.7 × 10^3^ FU than the three untreated scrRNAs at LP2000 control conditions with 36.7 ± 3.4 × 10^3^, 38.2 ± 5.0 × 10^3^, and 37.1 ± 3.2 × 10^3^ FU, respectively (*p* < 0.0001) (Figure 5D,F). Like LightSpot-FL-1, fluorescent microscopy images showed complete extinction of P-gp staining in SUM1315 cells and partial extinction in DU4475 cells following P-gp silencing (Figure 5D).

In contrast, for LightSpot-Scy-1, no extinction of fluorescent staining was detected after P-gp silencing in SUM1315 and DU4475 cells in microscopy images (Figure 5G). Indeed, untreated SUM1315 cells presented similar fluorescence intensity of 9.5 ± 0.8 × 10^3^ FU compared to siRNA-treated cells with 9.2 ± 1.4 × 10^3^ FU (*p* = 0.9293), scrRNA control cells with 9.3 ± 1.5 × 10^3^ FU (*p* = 0.9861), or LP2000 control cells with 9.5 ± 0.9 × 10^3^ FU (*p* = 0.9999) (Figure 5H). It was the same in the DU4475 cell line, for which no differences were observed between untreated control cell fluorescence of 21.7 ± 7.1 × 10^3^ FU, siRNA-treated cells with 21.1 ± 6.7 × 10^3^ FU (*p* = 0.9997), scrRNA-treated cells with 20.6 ± 5.5 × 10^3^ FU (*p* = 0.9843), and LP2000 control cells with 20.9 ± 6.3 × 10^3^ FU (*p* = 0.9975) (Figure 5I).

Finally, LightSpot-BdO-1 fluorescent staining intensity in SUM1315 P-gp silenced cells was greatly lower with 3.2 ± 0.8 × 10^3^ FU, compared to untreated, scrRNAs, and LP2000 control cells with 16.6 ± 1.5 × 10^3^, 17.5 ± 1.6 × 10^3^, and 17.3 ± 1.7 × 10^3^ FU, respectively (*p* < 0.0001) (Figure 5K). Likewise, after P-gp siRNA transfection, fluorescence of DU4475 cells was significantly reduced with 12.6 ± 0.3 × 10^3^ FU, compared to the three untreated, scrRNA, and LP2000 control conditions with 24.6 ± 2.7 × 10^3^, 23.2 ± 4.8 × 10^3^, and 23.2 ± 1.5 × 10^3^ FU, respectively (*p* < 0.0001) (Figure 5L). Thus, LightSpot-BdO-1 fluorescent staining was switched off completely in SUM1315 cells and partially in DU4475 after P-gp silencing (Figure 5J), like previously observed with LightSpot-FL-1 and LightSpot-BrX-1 compounds.

All these results suggest a high specificity of the three LightSpot-FL-1, LightSpot-BrX-1, and LightSpot-BdO-1 fluorescent compounds for the detection of the P-gp in both SUM1315 and DU4475 cell lines. In contrast, the LightSpot-Scy-1 compound does not seem to provide a specific P-gp staining, regarding the absence of staining reduction in P-gp knockdown cells.

### 2.3. Increased PARP-1 Inhibitor OLA Concentrations Impact on Lysosome Number in SUM1315 and DU4475 Cell Lines

In order to characterize lysosomal cell response to OLA treatment, SUM1315 and DU4475 cell lines were exposed to increasing concentrations of OLA, i.e., 10, 50, and 100 µM, during 3 h. Lysosomes were stained in both cell lines using anti-LAMP2 antibodies. For each OLA concentration tested, cell global LAMP2 staining intensity and lysosome number per cell were quantified in comparison with DMSO vehicle control.

**Figure 6:**
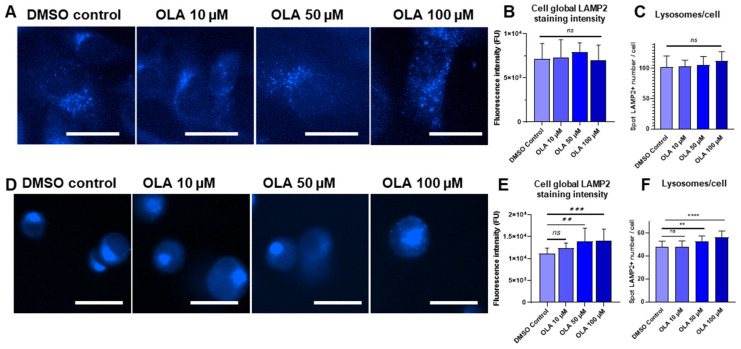
Impact of OLA treatment on lysosome number in SUM1315 and DU4475 cell lines. SUM1315 cells were exposed to 0 (DMSO control), 10, 50, and 100 µM OLA doses for 3 h. After treatment, the number of lysosomes per cell was analyzed for each OLA treatment dose. For this, anti-LAMP2 immunostaining was imaged using Cytation™5MV automate (BioTek^®^, M = 40x, DAPI filter, scale bar = 20 µm) for SUM1315 (**A**) and DU4475 cells (**D**). Mean cell fluorescence intensity was calculated with Gen5 software (BioTek^®^) for SUM1315 (**B**) and DU4475 (**E**) cells. The average number of lysosomes (LAMP2-positive spots) per cell was calculated using Icy software (2.4.2.0 version) for SUM1315 (**C**) and DU4475 (**F**) cells. Significance was determined by one-way ANOVA, ns *p* > 0.05, ** *p* < 0.01, *** *p* < 0.001, **** *p* < 0.0001.

In SUM1315 cells, fluorescence staining intensity was 7.0 ± 1.9 × 10^3^ FU for DMSO control, presenting no significant difference with 7.3 ± 1.4 × 10^3^, 7.8 ± 1.0 × 10^3^, and 7.0 ± 2.0 × 10^3^ FU for 10, 50, and 100 µM OLA doses, respectively (*p* = 0.9828, 0.4043, and 0.9743) (Figure 6B). In DU4475 cells, anti-LAMP2 fluorescence staining intensity was 11.1 ± 1.3 × 10^3^ FU for DMSO control and rose up to 12.3 ± 1.2 × 10^3^, 14.4 ± 3.5 × 10^3^, and 15.4 ± 3.0 × 10^3^ FU for 10, 50, and 100 µM OLA doses, respectively (*p* = 0.2579, 0.0017, and 0.0007) (Figure 6E). Otherwise, regarding lysosome number/cell using anti-LAMP2 immunostaining, in SUM1315 cells, no increase in lysosome number after OLA treatment was observed with an average of 102.3 ± 18.1 lysosomes per cell for the DMSO control condition, which is close to 103.7 ± 9.4 (*p* = 0.9861), 105.6 ± 14.2 (*p* = 0.8292), and 112.0 ± 15.8 (*p* = 0.0539) for 10, 50, and 100 µM OLA doses, respectively (Figure 6A,C). Interestingly, in DU4475 cells, OLA exposure induced an increase in the lysosome number. The average number of lysosomes per cell was 47.8 ± 5.2 for the DMSO control condition, which was close to the 48.2 ± 5.0 (*p* = 0.9874) observed for 10 µM OLA-exposed cells but lower than the 53.1 ± 4.4 (*p* = 0.0003, and 56.4 ± 5.4 (*p* = 0.0001) obtained for 50 and 100 µM OLA doses, respectively (Figure 6D,F).

### 2.4. Lysosomal-P-gp-Mediated Resistance in SUM1315 and DU4475 Cells After OLA Treatment

The implication of lysosomal P-gp in drug resistance mechanisms in both cell lines, SUM1315 and DU4475, was then studied after cells were exposed for 3 h to increasing concentrations of OLA. For this, the P-gp level at global cellular and lysosomal scales was quantified using the three LightSpot-FL-1, LightSpot-BrX-1, or LightSpot-BdO-1 fluorescent compounds. For this, cells were exposed for 3 h to either the DMSO vehicle control solution or 10, 50, or 100 µM of OLA. P-gp-mediated resistance was evaluated according to two complementary approaches. Indeed, (i) the determination of cell global P-gp mean fluorescence intensity and (ii) the number of bright fluorescent intracellular spots corresponding to lysosomal-P-gp were carried out.

**Figure 7:**
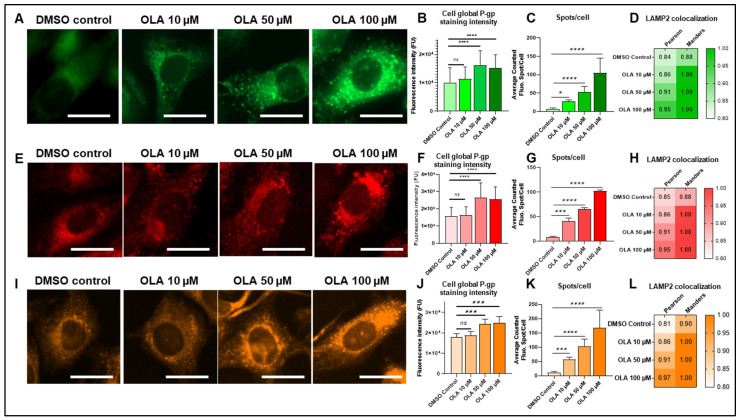
Impact of OLA treatment on lysosomal P-gp-mediated resistance in the SUM1315 cell line. SUM1315 cells were exposed to 0 (DMSO control), 10, 50, and 100 µM OLA doses for 3 h. After treatment, P-gp was stained with LightSpot fluorescent conjugates in PFA-fixed SUM1315 cells for each treatment condition. Fluorescent P-gp staining was acquired with Cytation™5MV (BioTek^®^, M = 40x, GFP, Cy5, and RFP filters, scale bar = 20 µm) cells for each LightSpot-FL-1 (**A**), LightSpot-BrX-1 (**E**), and LightSpot-BdO-1 (**I**) conjugate. Mean cell fluorescence intensity was calculated with Gen5 software (BioTek^®^) for LightSpot-FL-1 (**B**), LightSpot-BrX-1 (**F**), and LightSpot-BdO-1 (**J**). Fluorescence intensity data are presented on graphs as mean ± SD. Intracellular fluorescent spots of LightSpot staining were counted using Icy software. Average fluorescent spot number per cell is presented on graphs as mean ±  SD for LightSpot-FL-1 (**C**), LightSpot-BrX-1 (**G**), and LightSpot-BdO-1 (**K**) conjugates. Colocalization studies between LightSpot conjugates and anti-LAMP2 immunostaining were conducted for each treatment condition. For this, Pearson’s and Mander’s coefficients were calculated for LightSpot-FL-1 (**D**), LightSpot-BrX-1 (**H**), and LightSpot-BdO-1 (**L**) conjugates. Coefficients are presented as a heat map. Significance was determined by one-way ANOVA, ns *p* > 0.05, * *p* < 0.05, *** *p* < 0.001, **** *p* < 0.0001.

**Figure 8:**
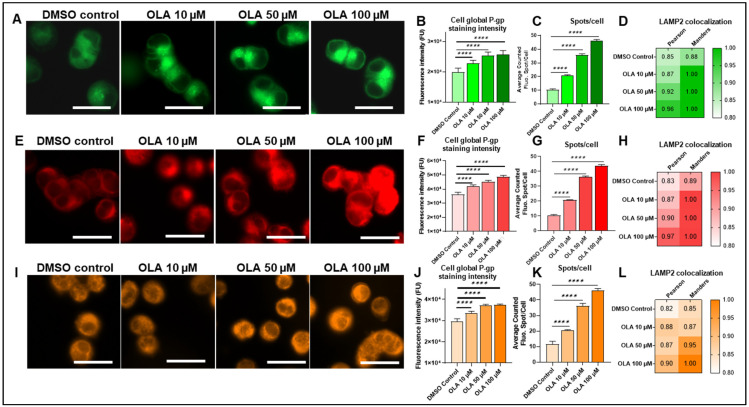
Impact of OLA treatment on lysosomal P-gp-mediated resistance in the DU4475 cell line. DU4475 cells were exposed to 0 (DMSO control), 10, 50, and 100 µM OLA doses for 3 h. After treatment, P-gp was stained with LightSpot fluorescent conjugates in PFA-fixed DU4475 cells for each treatment condition. Fluorescent P-gp staining was acquired with Cytation™5MV (BioTek^®^, M = 40x, GFP, Cy5, and RFP filters, scale bar = 20 µm) cells for each LightSpot-FL-1 (**A**), LightSpot-BrX-1 (**E**), and LightSpot-BdO-1 (**I**) conjugate. Mean cell fluorescence intensity was calculated with Gen5 software (BioTek^®^) for LightSpot-FL-1 (**B**), LightSpot-BrX-1 (**F**), and LightSpot-BdO-1 (**J**). Otherwise, intracellular fluorescent spots of LightSpot staining were counted using Icy software. Average fluorescent spot number per cell is presented on graphs as mean ±  SD for LightSpot-FL-1 (**C**), LightSpot-BrX-1 (**G**), and LightSpot-BdO-1 (**K**) conjugates. Colocalization studies between LightSpot conjugates and anti-LAMP2 immunostaining were conducted for each treatment condition. For this, Pearson’s and Mander’s coefficients were calculated for LightSpot-FL-1 (**D**), LightSpot-BrX-1 (**H**), and LightSpot-BdO-1 (**L**) conjugates. Coefficients are presented as a heat map. Significance was determined by one-way ANOVA, **** *p* < 0.0001.

Firstly, P-gp-mediated resistance was explored with LightSpot-FL-1. On fluorescence microscopy images, cellular fluorescent P-gp staining in SUM1315 and DU4475 appeared to be brighter with more fluorescent spots in the intracellular compartment in comparison to the DMSO non-treated control (Figure 7A and Figure 8A). In the SUM1315 cell line (Figure 7A), global P-gp cell fluorescence intensity was 10.1 ± 5.3 × 10^3^ FU for the DMSO control, which was close to 11.4 ± 4.3 × 10^3^ FU (*p* = 0.4072) for cells exposed to 10 µM OLA. In contrast, after cell exposure to 50 µM or 100 µM OLA, P-gp cell global intensity increased significantly compared to the control, with 16.2 ± 5.2 × 10^3^ FU (*p* < 0.00001) and 15.3 ± 4.6 × 10^3^ FU (*p* < 0.00001) (Figure 7B). For the DU4475 cell line, the 0.1% DMSO control presented a global fluorescence intensity of 19.9 ± 1.3 × 10^3^ FU, which was significantly lower than 22.8 ± 1.0 × 10^3^ FU (*p* < 0.00001), 25.4 ± 1.1 × 10^3^ FU (*p* < 0.00001), and 25.6 ± 1.4 × 10^3^ FU (*p* < 0.00001), obtained after cell exposure to 10, 50, and 100 µM of OLA, respectively (Figure 8B). Parallelly, the analysis of fluorescent intracellular spot number showed a clear, significant, dose-dependent increase in lysosomal P-gp after exposure to increasing OLA concentrations. Indeed, in SUM1315 cells, 7.5 ± 2.4 spots per cell for untreated control, 28.2 ± 3.4 spots per cell for OLA 10 µM (*p* = 0.0170), 52.3 ± 15.6 spots per cell for OLA 50 µM dose (*p* < 0.00001), and 104.5 ± 40.6 spots per cell for OLA 50 µM (*p* < 0.00001) were detected (Figure 7C). Similarly, in DU4475 cells, the spot number per cell was 10.6 ± 0.5 for the untreated control, 20.7 ± 0.6 spots for OLA at 10 µM (*p* < 0.00001), 35.8 ± 0.9 for OLA at 50 µM (*p* < 0.00001), and 46.0 ± 1.0 for OLA at 50 µM (*p* < 0.00001) (Figure 8C). Similarly, co-staining including LightSpot-FL-1 and lysosomal anti-LAMP-2 immunostaining was carried out after the same exposure times of SUM1315 and DU4475 cells with OLA treatment. Pearson’s coefficients in SUM1315 cells increased after treatment to 0.857, 0.905, and 0.952 for 10, 50, and 100 µM of OLA, respectively, compared to 0.835 for DMSO control (Figure 7D). Mander’s coefficients also increased, reaching 1.000 after 100 µM OLA exposure. In the same way, in DU4475 cells, colocalization was improved after OLA exposure, with Pearson’s coefficients increasing from 0.846 for the DMSO control to 0.867, 0.915, and 0.964 for 10, 50, and 100 µM of OLA, respectively (Figure 8D). Mander’s coefficients were also increased, reaching 1.000 for the three treatment concentrations.

Regarding P-gp-mediated resistance detected with LightSpot-BrX-1 fluorescent conjugate, an increase in fluorescence signal was detected on microscopy images across increasing doses of OLA treatment (Figure 7E and Figure 8E). In SUM1315 cells, global cell fluorescence of DMSO control was 12.1 ± 2.2 × 10^3^ FU, similarly to 10 µM OLA-treated cells with 12.1 ± 1.9 × 10^3^ FU (*p* = 0.9999). In contrast, this parameter increased to 20.9 ± 4.1 × 10^3^ FU (*p* < 0.00001) and 21.0 ± 3.8 × 10^3^ FU (*p* < 0.00001) after 50 µM and 100 µM OLA exposure, respectively (Figure 7F). DU4475 cells presented a more dose-dependent response with 36.3 ± 1.4 × 10^3^ FU for the DMSO control condition, lower than 42.0 ± 1.1 × 10^3^ FU (*p* < 0.00001), 45.2 ± 1.0 × 10^3^ FU (*p* < 0.00001), and 48.6 ± 1.1 × 10^3^ FU (*p* < 0.00001) for 10, 50, and 100 µM of OLA, respectively (Figure 8F). Otherwise, an analysis of the fluorescent intracellular spots number in SUM1315 cell line showed 8.1 ± 1.9 spots per cell for the untreated control. But this value increased significantly to 40.7 ± 6.5 (*p* < 0.00001), 65.1 ± 3.4 (*p* < 0.00001), and 102.4 ± 1.8 (*p* < 0.00001) spots per cell for 10, 50, and 100 µM OLA treatments, respectively (Figure 7G). Similarly, in DU4475 cells, fluorescent intracellular spot number increased across OLA doses, increasing from 10.4 ± 0.5 spots per cell for the untreated control to 20.6 ± 0.2 for OLA 10 µM (*p* < 0.00001), 36.1 ± 0.7 spots per cell for OLA 50 µM (*p* < 0.00001), and 43.5 ± 1.0 spots per cell for OLA 50 µM (*p* < 0.00001) (Figure 8G). As for LightSpot-FL-1, colocalization between P-gp staining and lysosomal staining was higher after OLA exposure. For SUM1315 cells, Pearson’s coefficients were 0.845 for the DMSO control and increased to 0.864, 0.912, and 0.945 for 10, 50, and 100 µM of OLA, respectively (Figure 7H). Likewise, in DU4475, Pearson’s coefficients increased from 0.831 for the vehicle control to 0.867, 0.904, and 0.969 for 10, 50, and 100 µM of OLA, respectively (Figure 8H). Mander’s coefficient reached 1.000 for several exposure conditions in both cell lines.

Finally, P-gp-mediated resistance was evaluated with LightSpot-BdO-1. Microscopy images showed a brighter fluorescent signal after OLA exposure than in the vehicle control (Figure 7I and Figure 8I). Indeed, in SUM1315 cells, global cell fluorescence was 17.9 ± 1.7 × 10^3^ FU for DMSO control, similarly to the OLA 10 µM dose with 18.8 ± 1.8 × 10^3^ FU (*p* = 0.1511). This parameter increased in the presence of OLA 50 µM, with 24.2 ± 2.3 × 10^3^ FU (*p* < 0.00001), and OLA 100 µM, with 25.1 ± 2.8 × 10^3^ FU (*p* < 0.00001) (Figure 7J). In DU4475 cells, global cell fluorescence was lower for the DMSO control with 29.5 ± 1.3 × 10^3^ FU than 33.6 ± 0.8 × 10^3^ FU (*p* < 0.00001), 37.1 ± 0.5 × 10^3^ FU (*p* < 0.00001), and 37.3 ± 0.4 × 10^3^ FU (*p* < 0.00001) measured for 10, 50, and 100 µM of OLA (Figure 8J). In complement, in SUM1315 cells, spot number per cell increased from 10.8 ± 3.8 for the untreated control, to 85.9 ± 33.1 for OLA 10 µM (*p* = 0.0002), to 102.9 ± 25.5 for OLA 50 µM (*p* < 0.00001), and to 168.1 ± 61.9 for OLA 100 µM (*p* < 0.00001) (Figure 7K). Similarly, in DU4475 cells, the DMSO control condition presented 11.6 ± 1.8 spots per cell for DMSO control and increased to 20.6 ± 0.3 (*p* < 0.00001), 36.3 ± 1.6 (*p* < 0.00001), and 46.3 ± 1.1 spots per cell for 10, 50, and 100 µM OLA (*p* < 0.00001), respectively (Figure 8K). As for the two previous fluorescent compounds, higher Pearson’s coefficient values were obtained in SUM1315 cells after OLA treatment exposure, with 0.864, 0.914, and 0.967 for 10, 50, and 100 µM OLA doses, respectively, compared to 0.812 for DMSO control (Figure 7L). DU4475 showed a similar Pearson’s coefficient increase of 0.878, 0.865, and 0.904 after 10, 50, and 100 µM OLA doses, respectively, compared to 0.816 for the DMSO control condition (Figure 8L). Overlap Mander’s coefficients were also higher after OLA exposure, attesting to a stronger colocalization of P-gp staining with lysosomes.

## 3. Discussion

It has been well established for several decades that plasma membrane P-gp plays a crucial role in the resistance of tumor cells to anticancer treatments [25,26]. This ATP-dependent transporter acts as a pump, recognizing a large panel of cytotoxic drugs, allowing their efflux out of cells, decreasing their intracellular accumulation, thus limiting drug efficacy. As a consequence, the P-gp overexpression represents a real obstacle in cancer treatments, increasing the rate of therapeutic failure [27]. Thus, the quantification of P-gp expression presents a great interest in (i) the understanding of resistance mechanisms, (ii) the development of new anticancer agents, and especially (iii) the clinical diagnosis of cancer resistance. In this regard, many efforts have been made since the 1990s to find a correlation between P-gp expression level and clinical outcomes. Several pieces of evidence allow us to associate high expression of the P-gp with unfavorable prognosis and shorter survival in solid tumors such as breast, ovarian, and lung cancers [6,28,29,30]. These encouraging findings even led us to consider the P-gp as a predictive marker of treatment response and prognosis [31,32]. However, studies on breast cancer showed that there was no link between P-gp expression and how patients responded to treatment. [33,34]. Moreover, some studies led to counterintuitive findings associating high P-gp expression with favorable prognosis on bladder, prostate, and esophageal cancer [35,36,37]. These contradictory results may explain why the P-gp expression level is not systematically evaluated during cancer diagnosis, despite the strong evidences of its implication in tumor resistance [38]. Nevertheless, chemoresistance remains a major issue in oncology. Indeed, around 80–90% of mortality in cancer patients is considered to be directly or indirectly attributed to drug resistance [39,40]. Nowadays, more studies are needed to explore the precise role of P-gp in tumor biology and clarify its relevance in clinical diagnosis. These investigations require efficient and standardized protocols for P-gp quantification. Indeed, almost all of the clinical works investigating P-gp expression levels were realized with antibody-based assays with epitope-dependent efficiency disparities. Since P-gp’s discovery about 50 years ago, newer methods have become available and will allow us to re-examine the interest of P-gp in cancer diagnosis [3]. In particular, imaging approaches will help to better investigate the localization of P-gp expression in biological samples [3]. In this context, our group has developed an innovative methodology based on small fluorescent molecules for the selective and specific localization and quantification of the P-gp in biological samples [19,21]. Indeed, we have conceived 24 P-gp-targeted fluorescent conjugates with different structures and optical properties [19]. These compounds were revealed to be an efficient method for quantifying P-gp overexpression in monolayer and spheroid cancer models after OLA treatment [19,21]. Moreover, they quickly penetrate cells and intracellular compartments to target the P-gp expressed at the cell membrane and organelle levels. Interestingly, our previous works showed that several of these compounds provided intracellular staining organized in filamentous or spot patterns. In the present study, intracellular P-gp staining is more deeply investigated using four LightSpot compounds.

Previous works revealed a much greater P-gp localization in organelles than on plasma membrane on leukemic and colorectal cancer cells [41,42]. Also, P-gp mRNA level was found to have a better correlation with the total (surface and intracellular) P-gp proteins than with only surface P-gp proteins [41]. These arguments confirm the importance of not only considering plasma membrane P-gp but also P-gp in the intracellular compartment, where it exercises a functional MDR activity, especially on lysosomes [43,44]. Indeed, P-gp has been described to be involved in the trapping of cytotoxic substrate drugs within lysosomal vesicles, preventing them from reaching their target and participating in cancer drug resistance [14,17]. To potentiate this effect, acidic lysosomal pH is responsible for the ionization of anticancer agents, hindering their diffusion through the lysosomal membrane and sequestering drugs in safe compartments. This unique lysosomal P-gp drug-trapping role is directly linked to autophagy, a catabolic pathway principally mediated by lysosomes, which is responsible for the degradation and the recycling of proteins and cellular components. In cancer, lysosomes and autophagy are described as playing a dual role. Indeed, autophagy is known to suppress tumorigenesis by inducing cell death, while it can also facilitate tumorigenesis by promoting tumor growth [45,46]. During therapeutic treatment, autophagy is reported to participate in the development of acquired resistance, especially in more aggressive tumoral subtypes, such as TNBC [47,48]. Several works demonstrated the high level of autophagy and lysosome number in TNBC [48,49]. This pathway plays a synergetic role with P-gp drug efflux in cancer resistance, like described in the TNBC MDA-MB-231 cell line [50].

Taken together, all these arguments highlight the crucial role of intracellular P-gp, especially when localized on lysosomes in aggressive cancer subtypes. Thereby, a total P-gp expression quantification, which encompasses both surface and intracellular P-gp, may represent a crucial element in cancer diagnosis to better characterize the disease and limit therapeutic failure rate. This evaluation must be adapted depending on cancer subtypes, according to a personalized medicine strategy. In this context, these works aimed to investigate the ability of four fluorescent conjugates, namely LightSpot-FL-1, LightSpot-BrX-1, LightSpot-Scy-1, and LightSpot-BdO-1, to detect and quantify P-gp-mediated OLA resistance and, in particular, resistance provided by lysosomal P-gp, in two SUM1351 and DU4475 TNBC cell models. Indeed, TNBC represents a pertinent model, considering the important challenges it involves in a clinical setting and its high propensity to develop MDR [51].

Firstly, our work focused on intracellular organelle staining, i.e., nucleus, mitochondria, and lysosomes, using the four selected fluorescent compounds. For this, co-staining was realized, with three organelles matching with the previously mentioned P-gp localization sites, namely the nucleus [52,53], mitochondria [15,16], and lysosome [13,54], but was not fully characterized. This approach is associated with a specificity evaluation of the P-gp detection in P-gp knockdown cells after P-gp siRNA transfection.

LightSpot-FL-1, LightSpot-BrX-1, and LightSpot-BdO-1 conjugates presented comparable staining distribution profiles with diffuse membrane-like and intense fluorescent spots, as previously demonstrated in SUM1315 and DU4475 TNBC cell line models [19,21]. Regarding Pearson’s and Mander’s coefficients, these stainings presented a strong colocalization with lysosomal immunostaining and no matches with mitochondrial or nuclear staining. Moreover, these three compounds presented a clear decrease in fluorescence intensity signal in P-gp knockdown SUM1315 and DU4475 cells. This extinction of LightSpot fluorescent compound staining intensity was complete in SUM1315 and partial in DU4475 for P-gp targeting. In parallel, the efficiency and specificity of P-gp silencing was also validated using anti-P-gp immunostaining in both SUM1315 and DU4475 cell lines (Figure S1). These results firstly highlight the efficiency of specific P-gp targeting by the three LightSpot-FL-1, LightSpot-BrX-1, and LightSpot-BDO-1 compounds. Secondly, they demonstrate a significant presence of the P-gp on the lysosomal membrane in both studied TNBC cell models, suggesting its role in cancer resistance.

Regarding the LightSpot-Scy-1 conjugate, a different cytosolic filamentous staining pattern that co-localized with mitochondrial immunostaining was detected. Otherwise, no colocalization with nuclear and lysosomal stainings was detected. However, this staining remains unchanged in P-gp knockdown SUM1315 and DU4475 cells, despite a validation of P-gp silencing in these cells. Thus, these results suggest a specific staining of LightSpot-Scy-1 conjugates in mitochondria, independently of the presence of the P-gp on this organelle. Indeed, in our previous works, we hypothesized that this conjugate is accumulated in the mitochondrial compartment [19]. For this reason, only the three specific P-gp-targeting conjugates, LightSpot-FL-1, LightSpot-BrX-1, and LightSpot-BdO-1, were used for the following experiments.

Then, the role of lysosomal P-gp in mediating anticancer drug resistance in both TNBC cell models was investigated. For this, firstly the cell global lysosomal quantity was evaluated in the presence of increasing concentrations of OLA using anti-LAMP2 immunostaining. In the SUM1315 cell line, the lysosomal quantity remained stable for 3 h, whereas in DU4475 this parameter increased slightly for the 50 and 100 µM OLA concentrations. Parallelly, the number of lysosomes for each cell line was quantified with or without OLA treatment. In the same way, in SUM1315 cells, the lysosome number remained stable across all tested OLA concentrations, while in DU4475 cells, a slight but significant increase in the lysosome number was detected in the presence of increased OLA concentrations. Similarly, other studies have reported that OLA treatment induces autophagy in cancer cells, but only with longer treatment durations [55,56].

Thereafter, lysosomal P-gp-mediated resistance was investigated in SUM1315 and DU4475 cell lines after OLA treatment. Firstly, P-gp expression level was quantified in the whole cell (including membrane P-gp and P-gp intracellular spots) using the three fluorescent compounds, i.e., LightSpot-FL-1, LightSpot-BrX-1, or LightSpot-BdO-1. Regardless of the fluorescent conjugate being used, a significant dose-dependent increase in P-gp expression was detected in both cell lines. Notably, the SUM1315 model exhibited a biphasic response in P-gp expression levels to OLA across all doses, while DU4475 cells demonstrated a progressive, dose-dependent increase in P-gp expression. Interestingly, the second approach we used, based on the detection and counting of fluorescent intracellular spots corresponding to lysosomal P-gp staining, provided more significant results. Indeed, with the three LightSpot conjugates and in both cell lines, the average number of P-gp-stained lysosomes per cell increased significantly across OLA doses. These results demonstrated a more impactful dose-dependent response with lysosomal P-gp compared to global cell fluorescence intensity. For example, after 100 µM OLA dose exposure, global cell fluorescence intensity analysis allowed us to measure an increase of 52%, while the quantification of fluorescent spot per cell showed an increase of 1293%. Moreover, the colocalization between P-gp-LightSpot-staining and LAMP2-lysosomal staining was carried out for each treatment condition, using the three LightSpot conjugates. Interestingly, both Pearson’s and Mander’s coefficients increased in correlation with increasing OLA concentrations, also revealing the recruitment of P-gp on lysosomes.

It was already described in prostate cancer cells that lysosomal-mediated cancer resistance impacted OLA response by the pre-activation of autophagy, promoting its degradation [56]. Otherwise, previous studies have investigated the response to different anticancer agents, noting that lysosomes are able to sequester ionizable P-gp substrates thanks to lysosomal acidity (pH~5), based on their basic pK_a_ values (pK_a_~8) [14,57]. Given that OLA has a low basic pK_a_ of 0.2, it is probably not ionized at lysosomal pH and, therefore, is not expected to be significantly trapped in lysosomes, as indicated by research conducted in rat hepatocytes [58]. Thus, further works investigating the distribution of OLA in cellular compartments will probably give additional responses on the lysosomal-P-gp response we have detected after OLA cell exposure. For this, the use of a fluorescent OLA derivative may be useful to visualize OLA distribution in cells [59].

These overall results clearly highlight the importance of investigating lysosomal P-gp in treatment resistance, in addition to analyzing the global P-gp cellular response. Notably, our results demonstrate that while OLA treatment induces a moderate increase in total cellular P-gp expression, the proportion of lysosomal P-gp exhibits a much stronger increase. These data suggest that the overall P-gp expression level within the cell does not fully account for the substantial increase in lysosomal P-gp obtained following OLA treatment. As previously described, this can probably be attributable to the recruitment of plasma membrane P-gp to lysosomes via the endocytosis process [17]. This hypothesis is notably supported by our colocalization study results. Indeed, they demonstrate that lysosomal anti-LAMP2 immunostaining and the three LightSpot fluorescent conjugates for P-gp staining exhibit a dose-dependent increase in colocalization coefficients following OLA exposure. All these results suggest that in the absence of anticancer treatment, P-gp is primarily localized at the cell membrane, where it functions to prevent the entry of potential chemotherapeutic agents into the cytoplasm. After OLA exposure, P-gp seems to be recruited at the lysosomal level, sequestering a portion of the drug that has already penetrated the intracellular compartment. This intriguing dual resistance mechanism, operating at both the membrane and lysosomal levels, will be the subject of our future investigations.

## 4. Materials and Methods

### 4.1. Fluorescent Compound Chemical Synthesis and Characterization

Fluorescent compounds were synthesized as previously described. Conjugates LightSpot-FL-1, LightSpot-BrX-1, LightSpot-Scy-1, and LightSpot-BdO-1 were designated in the reference article as compound **8**, **4**, **10**, and **12**, respectively [19] (Figure 1). Briefly, a common amine-bearing P-gp inhibitor derivative was reacted with commercial NHS ester fluorochromes, BODIPY FL, BODIPY 650/665 X, sulfocyanine 3, and BODIPY 564/570 (Interchim, Montlucon, France) to yield LightSpot-FL-1, LightSpot-BrX-1, LightSpot-Scy-1, and LightSpot-BdO-1, respectively. After semi-preparative HPLC-UV-DAD purification, compounds were fully characterized by ^1^H NMR, ^13^C NMR, and high-resolution mass spectrometry. All compounds were stored as dry aliquots at −20 °C and dissolved in DMSO (1.0 mM stock solutions) for biological experiments.

### 4.2. Cell Culture

SUM1315 (MO2, Asterand, Detroit, MI, USA) and DU4475 (ATCC^®^, HTB-123™) cell lines were authenticated with STR/DNA profiling (Eurofins Genomics, Nantes, France). SUM1315 cells were cultured in Ham’s F-12 medium (catalog no. 21765037, Gibco, Dublin, Ireland), supplemented with 5% FCS (Fetal Calf Serum, Eurobio Scientific, Paris, France), HEPES (10 mM, catalog no. H0887, Sigma Aldrich, Saint-Louis, MO, USA), 10 ng/mL epidermal growth factor (EGF, catalog no. E9644, Sigma Aldrich, Saint-Louis, MO, USA), insulin (4 µg/mL, catalog no. F56X377, Novo Nordisk, Bagsværd, Denmark), and 20 µg/mL gentamicin (Panpharma, Paris, France). DU4475 cells were cultured in RPMI 1640 medium (catalog no. 31870074, Gibco, Dublin, Ireland) supplemented with 10% FCS and 20 µg/mL gentamicin. Both cell lines were maintained in a humid atmosphere (37 °C with 5% CO_2_).

### 4.3. Fluorescent Compound Cellular Staining

Cellular stainings with fluorescent compounds were carried out according to the protocol optimized in previous works [19,21]. SUM1315 adherent cells were seeded in IbiTreat 8-well µ-Slides (Ibidi^®^, Gräfelfing, Germany) at 50 × 10^3^ cells per well. After 24 h, cells were fixed in 4% paraformaldehyde (PFA) solution for 10 min at 4 °C. DU4475 in suspension cells were directly harvested from flask culture to have 1 million cells per experimental condition and fixed according to the same protocol. Cells were then incubated with the fluorescent compounds diluted in PBS at 1 µM for one hour. At the end of the staining, cells were washed for 20 min with PBS three times. Observations were made with a Cytation™3 MV fluorescence microscopy module (BioTek^®^, Winooski, VT, USA) equipped with GFP (excitation 469 nm; emission 525 nm), RFP (excitation 531 nm; emission 593 nm), and Cy5 (excitation 628 nm; emission 685 nm) fluorescence filters. Global cell mean fluorescence intensity was calculated with Gen5 3.08 software.

### 4.4. P-gp Knockdown Using Specific siRNAs

SUM1315 and DU4475 cells were seeded in 24-well plates at 30 × 10^3^ cells per well. The day after, P-gp knockdown was realized by exposing cells with specific P-gp siRNAs (catalog no. AM51331, assay ID #4123, Invitrogen, Waltham, MA, USA) diluted at 50 nM in a culture medium and Lipofectamine 2000 (1:400, catalog no. 11668019, Thermo Fisher, Waltham, MA, USA) solution over 72 h. siRNA sense sequence was 5′ GGAUAUUAGGACCAUAAAU 3′. As controls, cells exposed with no treatment, with scrambled siRNAs (scr siRNA, 50 nM, catalog no. AM4611, Invitrogen, Waltham, MA, USA), or with Lipofectamine 2000 (1:400) alone were also analyzed.

### 4.5. Subcellular and Colocalization Studies

For subcellular distribution studies, co-staining was realized, including fluorescent compound staining, and Hoescht33258, anti-LAMP2, or anti-ATP5A, to localize nuclei, lysosomes and mitochondria, respectively. For nuclear staining, PFA-fixed cells were exposed with Hoescht33258 (1 µg/mL, catalog no. 14530, Sigma Aldrich, Saint-Louis, MO, USA) for 10 min. For lysosomal staining, after BSA blocking and Triton (1:200, catalog no. T8532 Sigma Aldrich, Saint-Louis, MO, USA) membrane permeabilization, cells were incubated with anti-LAMP2 antibodies (1:250, catalog. ab25631, Abcam, Cambridge, UK). Cells were then incubated with AlexaFluor™350 Goat anti-Mouse antibodies (1:800, catalog no. A11045, Invitrogen, Waltham, MA, USA) for one hour. For mitochondrial staining, after BSA blocking and Triton membrane permeabilization, cells were incubated with anti-ATP5A antibodies (1:500, catalog no. ab14748, Abcam, Cambridge, UK). Cells were then incubated with AlexaFluor™350 Goat anti-Mouse antibodies (1:800, catalog no. A11045, Invitrogen, Waltham, MA, USA) for one hour. P-gp staining with fluorescent compounds was executed lastly to preserve staining fluorescence intensity, as previously described. Observations were made with the Cytation™3 MV fluorescence microscopy module, equipped with DAPI (excitation 377 nm; emission 447 nm), GFP (excitation 469 nm; emission 525 nm), RFP (excitation 531 nm; emission 593 nm), and Cy5 (excitation 628 nm; emission 685 nm) fluorescence filters. Global cell mean fluorescence intensity was calculated with Gen5 3.08 software. Colocalization studies were conducted between fluorescent compound staining images and organelle staining images. For this, Pearson’s correlation coefficient and Mander’s coefficient were determined using the JACoP plugin on ImageJ 1.53t software (National Institutes of Health, Bethesda, MD, USA).

### 4.6. OLA Exposure and P-gp Induction Analysis

OLA (catalog no. FO33122, Carbosynth, Compton, CA, USA) was solubilized in DMSO to prepare a 100 mM stock solution. Dilutions were prepared in complete cell culture medium for final treatment concentrations of 10, 50, and 100 µM. The final DMSO concentration remained constant at 0.1% in all tested conditions. For OLA exposure experiments, SUM1315 and DU4475 cells were seeded in 24-well plates at 60 × 10^3^ cells per well. Cells were exposed for 3 h with the three OLA concentrations in normal culture conditions (humid atmosphere, 37 °C, with 5% CO_2_). At the end of the exposure, cells were directly fixed in 4% PFA solution for 10 min at 4 °C. P-gp staining with fluorescent compounds was realized as previously described for different experimental conditions. Cells were observed with the Cytation™3 MV fluorescence microscopy module, equipped with GFP (excitation 469 nm; emission 525 nm), RFP (excitation 531 nm; emission 593 nm), and Cy5 (excitation 628 nm; emission 685 nm) fluorescence filters. Global cell mean fluorescence intensity was calculated with Gen5 3.08 software. In complement, spot detector analysis was conducted on fluorescent compound staining images, using Icy software (Institute Paster, Paris, France), in order to evaluate the number of lysosomes presenting P-gp in their membrane per cell. Similar protocols were applied after OLA exposure, detecting lysosomes with anti-LAMP2 immunostaining, as previously described.

### 4.7. Statistical Analysis

Results are presented as mean ± standard deviation. All experiments were performed independently on at least three separate occasions. One-way ANOVA or non-parametric Kruskal–Wallis tests, associated with Tukey’s multiple mean comparison tests, were used to evaluate statistical significance. Results were considered statistically different when *p* < 0.05. Differences were noted as follows: *p* < 0.05 (*), *p* < 0.01 (**), *p* < 0.001 (***), *p* < 0.0001 (****) and *p* < 0.00001 (*****). Non-significant results were noted as “ns”.

## 5. Conclusions

In conclusion, this study aimed to elucidate the critical role of lysosomal P-gp in mediating OLA resistance using four fluorescent conjugates. Our results demonstrate that in both SUM1315 and DU4475 TNBC cell models, LightSpot-FL-1, LightSpot-BrX-1, and LightSpot-BdO-1 specifically target the P-gp and exhibit substantial colocalization with lysosomal markers. In contrast, LightSpot-Scy-1 is localized in the mitochondria and does not exhibit specificity for P-gp. Furthermore, we evaluated the lysosomal P-gp-mediated resistance in response to OLA treatment. Our findings reveal that OLA exposure significantly enhances P-gp expression levels and the distribution of P-gp on lysosomes. All these results suggest that P-gp is used by the lysosomes to sequester the drug, highlighting a dual resistance mechanism involving both membrane and lysosomal P-gp. These findings underscore the crucial role of lysosomal P-gp and emphasize the necessity of assessing its involvement in drug-resistance mechanisms.

## 6. Patents

These present study refers to the international patent filing # PCT/EP2020/077694 from 2 October 2020.

## Data Availability

All data are contained within the manuscript.

## Supplementary Materials

The following supporting information can be downloaded at https://www.mdpi.com/article/10.3390/ijms26146675/s1.

## Abbreviations

The following abbreviations are used in this manuscript:

ABC	ATP Binding Cassette
BSA	Bovine Serum Albumin
FCS	Fetal Calf Serum
FU	Fluorescence Unit
HPLC-UV-DAD	High Performance Liquid Chromatography-Ultra Violet-Diode Array Detector
MDR	Multidrug Resistance
OLA	Olaparib
PFA	Paraformaldehyde
P-gp	Permeability Glycoprotein
TNBC	Triple-Negative Breast Cancer
