# Supplementary material for: LightSpot Fluorescent Conjugates as Highly Efficient Tools for Lysosomal P-gp Quantification in Olaparib-Treated Triple-Negative Breast Cancer Cells
Source: Int J Mol Sci. 2025 Jul 11;26(14):6675. doi: 10.3390/ijms26146675 (PMC12294673; doi:10.3390/ijms26146675)
Supplement: Supplementary file 1 [file ijms-26-06675-s001.zip › ijms-3748310-supplementary.pdf]

LightSpot fluorescent conjugates as highly efficient tools for lysosomal P-gp quantification in Olaparib-treated Triple-Negative Breast Cancer cells

Antoine Goisnard[1], Pierre Daumar[1], Maxime Dubois[1], Elodie Gay[1], Manon Roux[1], Marie Depresle[1], Frédérique Penault-Llorca[2], Emmanuelle Mounetou[1], Mahchid Bamdad[1,*]

From the [1] Université Clermont Auvergne, Institut Universitaire de Technologie, UMR INSERM-UCA, U1240, Imagerie Moléculaire et Stratégies Théranostiques, F-63000 Clermont Ferrand, France ; [2] Université Clermont Auvergne, Centre de Lutte Contre le Cancer Jean Perrin, UMR INSERM-UCA, U1240, Imagerie Moléculaire et Stratégies Théranostiques, F-63000 Clermont Ferrand, France

* For correspondence: mahchid.bamdad@uca.fr.

List of supplementary information:

Figure S1: Efficiency and specificity validation of P-gp siRNAs to obtain P-gp knock-downed cell models

Figure S2: Cell viability evaluation after OLA 3 hours exposure

Figure S3: Evaluation of OLA drug sensitivity in SUM1315 and DU4475 cell models

Figure S4: Imaging controls with unbounded fluorophores

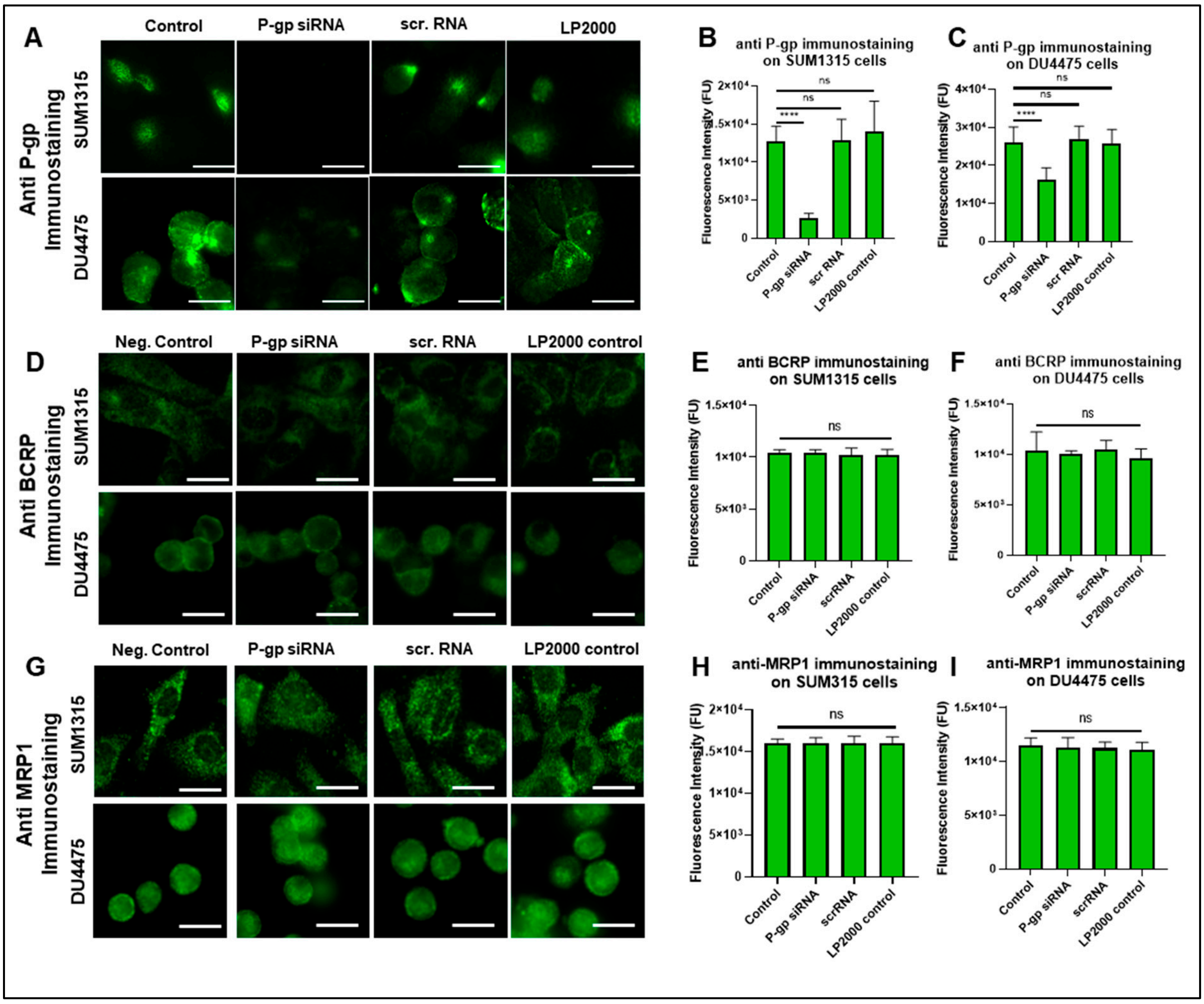

Figure S1: Efficiency and specificity validation of P-gp siRNAs to obtain P-gp knock-downed cell models. *SUM1315 and DU4475 cells were exposed for 72 hours with either no additional reagent (control), P-gp siRNAs, scrambled RNAs (scr RNA), or Lipofectamine2000 alone (LP2000). Specific anti-P-gp (**A**), anti-BCRP (**D**), and anti-MRP1 (**G**) immunostainings were then conducted in PFA fixed cells with the following primary antibodies: anti-P-gp clone F4 antibody (1:75, catalog no. MA5-13854, Thermo Fisher, Waltham, Massachusetts, USA), anti-Clone P-gp C494 (1:20, cat. no. ALX-801-003-C100, EnzoLife, Lausen, Switzerland), anti-BCRP antibody clone 5D3 (1:200, cat. no. cat. MAB4155, Sigma Aldrich, St Louis, Missouri, United States), or an anti-MRP1 antibody clone mrpm5 (1:50, cat. no ab24102, Abcam, Cambridge, UK). For immunostaining with antibody clones C494, BXP21, and mrpm5 targeting intracellular epitopes, cells were first permeabilized by incubating them in 0.3% triton solution for 10 min. Cells were incubated in bovine serum albumin blocking solution (BSA, 1%) for 1 hour before being exposed to the primary antibody for an additional hour. Cells were then incubated for 1 hour with AlexaFluor™ 488 goat anti-mouse secondary antibodies (1:800, catalog no. A11001, Invitrogen, Waltham, MA, USA). The stained cells were examined using the Cytation™3 MV fluorescence microscopy module, equipped with a GFP fluorescence filter (excitation 469 nm; emission 525 nm). Mean fluorescence intensity was calculated using Gen5 software (BioTek®) in SUM1315 (**B, E, & H**) and DU4475 (**C, F, & I**) cells. Data are presented on graphs as mean± SD. Significance was determined by one-way ANOVA, ns $p > 0.05$, ****$p < 0.0001$.*

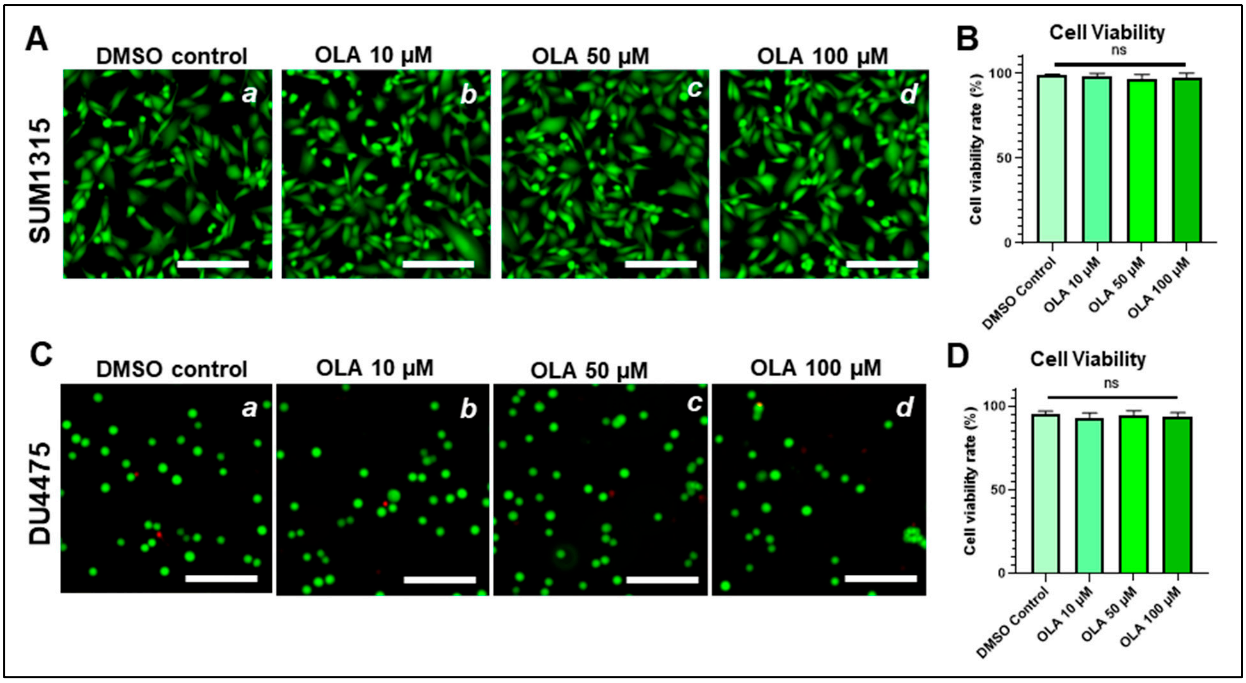

Figure S2: Cell viability evaluation after OLA 3 hours exposure. *SUM1315 and DU4475 cells were exposed with 0 (DMSO control), 10, 50, and 100 µM OLA doses for 3 hours. After treatment cell viability was evaluated using Live/Dead fluorescence-based tests. For this, images were acquired for each dose with Cytation™5MV (BioTek®, M=10x, GFP and IP filters, scale bare=200 µm) in SUM1315 (**A**) and DU4475 (**B**) cells. Cell viability rate was calculated with Gen5 software (BioTek®) for both SUM1315 (**E**) and DU4475 (**G**) cell lines.*

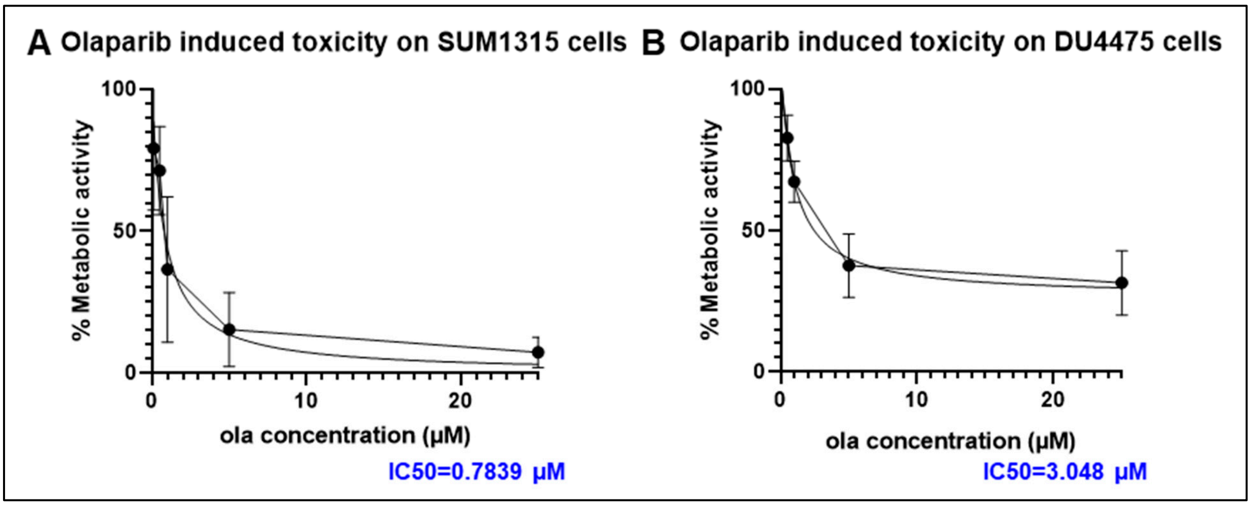

Figure S3: Evaluation of OLA drug sensitivity in SUM1315 and DU4475 cell models. *Both cell lines were exposed during 96 hours with increasing ola doses. After treatment, supernatant medium was gently removed and cells were incubated for 30 minutes in 60 µM resazurin solution (catalog no. 189900010, ACROS Organics, Waltham, MA, USA). Cell metabolic activity was evaluated with Resorufin concentration determined with resorufin fluorimetric quantification using Cytation™5MV (BioTek®, 593 nm)***.*** *Normalized metabolic activity data are presented on graph as mean± SD for SUM1315 (**A**) and DU4475 (**B**) cell lines. IC50 values for each cell line were estimated using GraphPad software (Prism®) non-linear regression analysis.*

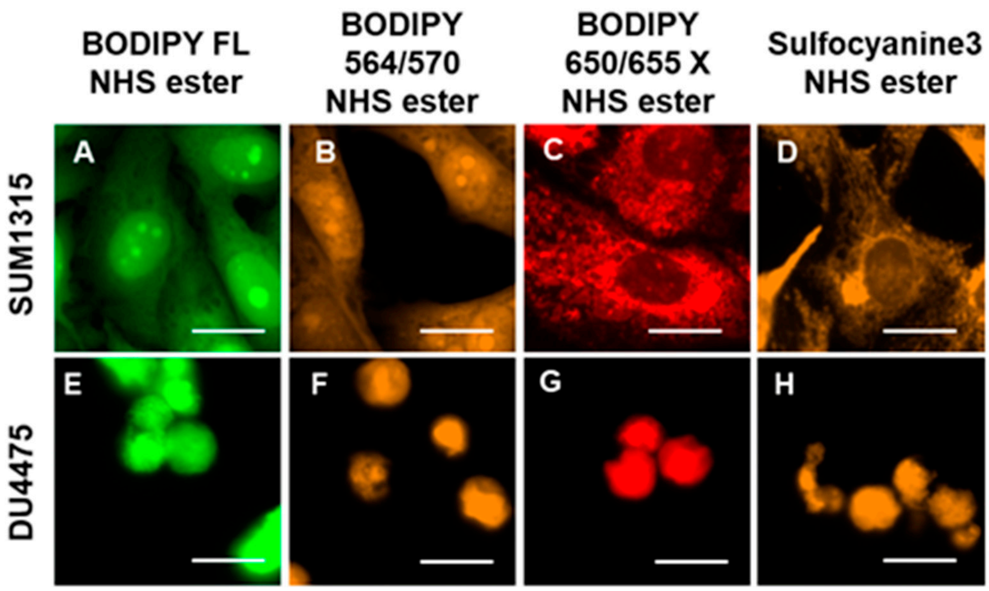

Figure S4: Imaging controls with unbounded fluorophores*. Intracellular distribution studies were performed by fluorescence imaging in SUM1315 and DU4475 cells with the four commercial NHS ester fluorophores BODIPY FL, BODIPY 564/570, BODIPY 650/665 X, and sulfocyanine 3. PFA-fixed cells were incubated with a fluorophore at 1 µM for 1h and rinsed 3 times with PBS before imaging withCytation™3 MV (BioTek®, USA). Images show characteristic staining patterns. (****A, E****) BODIPY FL staining pattern (green). (****B, F****) BODIPY 564/570 staining pattern (orange). (****C, G****) BODIPY 650/665 X staining pattern (red). (****D, H****) sulfocyanine 3 staining pattern (orange).*
